# Surfactant Protein-A Modulates LPS-Induced TLR4 Localization and Signaling *via* β-Arrestin 2

**DOI:** 10.1371/journal.pone.0059896

**Published:** 2013-03-25

**Authors:** Vicky Sender, Linda Lang, Cordula Stamme

**Affiliations:** 1 Division of Cellular Pneumology, Research Center Borstel, Leibniz-Center for Medicine and Biosciences, Borstel, Germany; 2 Department of Anesthesiology, University Hospital of Lübeck, Lübeck, Germany; University of Bern, Switzerland

## Abstract

The soluble C-type lectin surfactant protein (SP)-A mediates lung immune responses partially *via* its direct effects on alveolar macrophages (AM), the main resident leukocytes exposed to antigens. SP-A modulates the AM threshold of lipopolysaccharide (LPS) activity towards an anti-inflammatory phenotype both *in vitro* and *in vivo* through various mechanisms. LPS responses are tightly regulated *via* distinct pathways including subcellular TLR4 localization and thus ligand sensing. The cytosolic scaffold and signaling protein β-arrestin 2 acts as negative regulator of LPS-induced TLR4 activation. Here we show that SP-A neither increases TLR4 abundancy nor co-localizes with TLR4 in primary AM. SP-A significantly reduces the LPS-induced co-localization of TLR4 with the early endosome antigen (EEA) 1 by promoting the co-localization of TLR4 with the post-Golgi compartment marker Vti1b in freshly isolated AM from rats and wild-type (WT) mice, but not in β-arrestin 2^−/−^ AM. Compared to WT mice pulmonary LPS-induced TNF-α release in β-arrestin 2^−/−^ mice is accelerated and enhanced and exogenous SP-A fails to inhibit both lung LPS-induced TNF-α release and TLR4/EEA1 positioning. SP-A, but not LPS, enhances β-arrestin 2 protein expression in a time-dependent manner in primary rat AM. The constitutive expression of β-arrestin 2 in AM from SP-A^−/−^ mice is significantly reduced compared to SP-A^+/+^ mice and is rescued by SP-A. Prolonged endosome retention of LPS-induced TLR4 in AM from SP-A^−/−^ mice is restored by exogenous SP-A, and is antagonized by β-arrestin 2 blocking peptides. LPS induces β-arrestin 2/TLR4 association in primary AM which is further enhanced by SP-A. The data demonstrate that SP-A modulates LPS-induced TLR4 trafficking and signaling *in vitro* and *in vivo* engaging β-arrestin 2.

## Introduction

The soluble human pulmonary C-type lectin, surfactant protein (SP)-A, is the most abundant surfactant-associated protein in the lung with important functions in lung homeostasis *in vivo* [1]–[3]. SP-A avidly binds to alveolar macrophages (AM), the major resident phagocytic cell in the alveolar space, and specifically modifies their phenotype and function both constitutively and in response to Gram-negative bacteria or isolated LPS (endotoxin). LPS is a highly potent stimulus of the human immune system *via* the TLR4/MD2 receptor complex [4]. Although TLR4 is essential for initiating activation of innate defenses, excessive inflammation in response to prolonged activation can prove detrimental. As such, regulatory mechanisms have developed to avoid inappropriate inflammatory responses. The cellular responsiveness to LPS is in part regulated by the cell surface membrane levels of TLR4 that are determined by the amount of TLR4 trafficking from the Golgi to the plasma membrane and the amount of TLR4 internalized into endosomes [5]. TLR4 translocates to the cell surface upon LPS exposure and the LPS/TLR4/MD2 complex is internalized into early endosomes and then delivered to recycling endosomes and Golgi for recycling or to lysosomes for degradation 6–9. In a sequential manner TLR4 utilises specific adaptor proteins at the plasma membrane and in early endosomes activating the NF-κB pathway and the type 1 interferon pathway, respectively [5]. Although compartmentalized TLR4 signaling has been extensively studied, it is largely unknown whether cell-type-specific responses to LPS are dictated by cell specific differences in subcellular positioning of TLR4 [10], [11]. Further, the role of tissue-specific microenvironmental factors in regulating intracellular TLR4 localization remains poorly defined.

SP-A modulates the threshold of LPS activity towards an anti-inflammatory phenotype both *in vitro* and *in vivo* through distinct mechanisms. Inhibition of LPS-induced NF-κB activity by SP-A has been described to occur *via* direct interaction of SP-A with LPS itself [12] or components of the LPS receptor complex, including CD14 [13], [14], MD-2 [15], and TLR4 [15], but also independently of the LPS-receptor complex [16], [17]. We have previously demonstrated that SP-A specifically and transiently modulates endocytic/phagocytic membrane trafficking in primary AM functionally enhancing the lysosomal delivery of GFP-*E. coli* in these cells [18]. However, it is unknown whether SP-A-mediated alterations in AM membrane trafficking can also specifically coordinate TLR4 subcellular localization and function in AM.

The multifunctional adaptor and signaling proteins β-arrestin 1 and β-arrestin 2 are expressed throughout the human body with the expression of β-arrestin 2 being enriched in macrophages [19]. β-arrestin 1/2double-knockout mice are neonatal lethal due to respiratory stress based on lung immaturity including decreased surfactant generation and significantly lowered SP-A expression [20]. The fact, however, that selectively β-arrestin 1^−/−^ or β-arrestin 2^−/−^ mice are viable implies that β-arrestins can substitute for each other to some extent in mediating major developmental effects on lung maturation. In contrast, data obtained from systemic mouse infection models suggest, that β-arrestin 1 cannot substitute for β-arrestin 2 in the lung [21], [22]. Whereas the classical function of β-arrestins in regulating desensitization of G-protein coupled receptors is established, their role in regulating signaling and/or endocytosis of structurally unrelated receptors, such as TLRs as well as their function as activating scaffolds for signaling pathways is only beginning to be revealed [23]. In the systemic murine caecal ligation and puncture model, the survival rate of β-arrestin 2^−/−^ mice is significantly decreased compared to WT mice with increased plasma IL-6 activity, more severe lung damage and higher bacterial loads [21]. These findings support previous *in vivo* studies showing that intraperitoneally administered LPS induced higher mortality in β-arrestin 2^−/−^ mice, mechanistically related to suppressed signaling to NF-κB by direct β-arrestins/TRAF6 binding [24]. In addition, β-arrestin 2 directly interacts with the predominant NF-κB inhibitor IκB-α preventing its phosphorylation and degradation [25], [26]. Collectively, these studies suggest that β-arrestin 2 is a pivotal negative regulator of TLR4-dependent cytokine production and immune responses. Although there is strong evidence for cell and environment specificity of β-arrestin mode of action, the role of β-arrestin 2 in lung-specific responses to LPS *in vivo* has not yet been defined.

In this study, we demonstrate that SP-A affects the spatiotemporal compartmentalization and signaling of LPS-induced TLR4 in primary AM. In addition, we show that β-arrestin 2 is critically involved in SP-A-mediated inhibition of TLR4 translocation and signaling *in vitro* and *in vivo*.

## Materials and Methods

### Animals and Ethics Statement

Primary AM were obtained from pathogen-free male Sprague-Dawley rats, from C57BL/6J WT mice (Charles River Laboratories), from SP-A^−/−^ mice generated as described before [27], from β-arrestin2^−/−^ mice kindly provided by Dr. Robert Lefkowitz (Duke University) [28] and the corresponding WT C57BL/6 mice (Charles River Laboratories) as described previously [29]. Animal care and experiments were approved by the Schleswig-Holstein Ministry of Environment, Nature, and Forestation (Permit Number: V 312-72241.123-3) and all efforts were made to minimize suffering. All mice used were between 6 and 12 weeks of age and were maintained at the Research Center Borstel animal facility under specific pathogen-free conditions.

### Reagents

The smooth LPS from *Salmonella friedenau* strain H909 was extracted by the phenol/water method, purified, lyophilized, and transformed into the triethylamine salt form [30]. RPMI 1640 medium, FBS, and Dulbecco's PBS were from PAA Laboratories GmbH. The bicinchoninic acid reagent was from Interchim. Beta-arrestin 2 blocking peptides, mouse anti-β-actin, anti-β-arrestin 2, rabbit anti-EEA1, goat anti-TLR4 (for confocal microscopy), rabbit anti-IκB-α, and HRP-conjugated goat anti-rabbit and donkey anti-mouse IgG were from Santa Cruz Biotechnology; Rabbit anti-TLR4 (for Western blot) and Dynabeads® were from Invitrogen. Amine-reactive Alexa Fluor 555 carboxylic acid, succinimidyl ester, DAPI, Alexa Fluor 488 donkey anti-goat IgG and Alexa Fluor 633 goat anti-rabbit IgG were from Molecular Probes (Invitrogen). The mouse anti-Vti1b (vesicle transport through interaction with t-SNAREs homologue 1B) antibody was from BD Biosciences. All other reagents (except as noted) were obtained from Carl Roth GmbH & Co. KG.

### SP-A purification

Human SP-A was purified from bronchoalveolar lavage of patients with alveolar proteinosis, as described in detail elsewhere [31]. Briefly, the lavage fluid was treated with butanol to extract SP-A, and the resulting pellet was sequentially solubilized in octylglucoside and 5 mM Tris-buffered water (pH 7.4). To reduce endotoxin contamination, SP-A was treated with polymyxin B agarose beads. SP-A preparations were tested for the presence of bacterial endotoxin using a *Limulus* amebocyte lysate assay (Pyroquant Diagnostic, Mörfeld-Walldorf, Germany). All SP-A preparations used contained <0.2 pg endotoxin/µg SP-A.

### SP-A labelling

Purified SP-A was labeled with amine-reactive Alexa Fluor 555 carboxylic acid, succinimidyl ester as described previously [18]. Briefly, SP-A was incubated with the respective conjugate in 100 mM sodium bicarbonate buffer (pH 9.0) for 1 h at room temperature. The reaction was stopped by adding 300 mM hydroxylamine hydrochloride (pH 8.5) and dialyzed overnight in a QuixSep Micro Dialyzer against PBS to remove unbound conjugate. Labeled proteins were analyzed by SDS-PAGE with subsequent Western Blot and Coomassie staining, and functionality of labeled SP-A was compared to that of unlabeled protein as shown previously [32].

### Intratracheal application

Gender matched β-arrestin 2^−/−^ mice and corresponding WT mice (6–12 weeks old) were challenged intratracheally (i. tr.) with 2.5 µg/kg BW (0.05 µg/animal) of LPS or 2.5 µg/kg BW of LPS plus 5 mg/kg BW (100 µg/animal) of SP-A dissolved in a total volume of 100 µl sterile PBS using a Small Animal Laryngoscope-Model LS-2 and a MicroSprayer® Aerosolizer-Model IA-1C (Penn-Century. Inc. Wyndmoor, PA). Control mice were instilled i. tr. with 100 µl LPS-free sterile PBS. For intratracheal applications, all animals were anesthetized by i. p. injection of ketamin (WDT, Garbsen, Germany; 100 mg/kg body weight (BW) and xylazine (Bayer AG, Leverkusen, Germany; 10 mg/kg BW) mixture. After 60, 120, 240, or 300 min mice were euthanized by i. p. injection of a lethal dose of Nembutal (pentobarbital sodium), blood samples were collected by heart puncture and bronchoalveolar lavage (BAL) was performed. BAL fluid was centrifuged at 400× g for 10 min and the volume of cell-free lavage fluid recovered was measured for each animal. The samples were stored in aliquots in sterile 1.5-ml microfuge tubes at −80°C until assayed for TNF-α concentration. Cells recovered from BAL were used to prepare whole cell lysates, cytosolic fractions, and for confocal microscopy. Cytosolic extracts were prepared as described before [29] and the protein concentration was determined by bicinchoninic acid assay (BCA) using the BC Assay Protein Quantification Kit (Interchim, Montluçon, France).

### Stimulation of primary AM

Primary AM from rats and mice were isolated as described previously [29], [33]. Rat AM were seeded in 24-well plates, and allowed to attach for 90 min at 37°C in a 5% CO_2_ atmosphere. AM from SP-A^−/−^ mice, β-arrestin2^−/−^ mice, and the corresponding WT mice were dispensed at a density of 2×10^5^/500 µl per tube (Protein LoBind 1.5 ml microfuge tubes; Eppendorf, Hamburg, Germany) in the presence of 0.2% HI-FBS, and experiments were carried out with non-adherent soluble cells. The cells were left untreated or treated with SP-A (40 µg/ml), LPS (100 ng/ml), or both for indicated times at 37°C in the presence of 0.2% HI-FBS. After stimulation, cells were washed with 500 µl of cold PBS, scraped off, and lysed in Laemmli-buffer for 30 min at 4°C to prepare whole cell lysates.

### Co-Immunoprecipitation

Rat AM (2×10^6^/well) were left untreated or treated with LPS (100 ng/ml), SP-A (40 µg/ml), or both for indicated times followed by lysis in 500 µl RIPA-buffer (50 mM Tris-HCl, 150 mM NaCl, 1% NP-40, pH 8.0) for 30 min at 4°C. Anti-β-arrestin 2 (5 µg/ml) were preincubated with Dynabeads for 60 min at RT with gentle agitation. Then, 100 µg of the protein lysates were incubated with the beads/Ab-complex over night at 4°C with gentle agitation, and were washed three times with PBS. Immune complexes were released by heating the beads for 5 min at 95°C with sample buffer. Subsequently, 0.5 M DTT and bromphenol blue were added and Western analysis on TLR4 was performed.

### Western analysis

Western analysis was performed on cytosolic fractions, whole cell lysates and immunoprecipitated samples. Lysates were separated on SDS-PAGE, and transferred to nitrocellulose membrane. Membranes were incubated with anti-TLR4 (rabbit polyclonal; 1∶40), anti-β-arrstin 2, or anti-β-actin (all mouse monoclonal; 1∶200), or rabbit anti-IκB-α (1∶700) Abs. Goat anti-rabbit IgG-HRP or donkey anti-mouse IgG-HRP (both 1∶2000) served as secondary Abs. Immunoreactive proteins were visualized using the ECL Western blotting detection system, band intensity was quantified by analysis with ImageJ 1.42 (NIH), and data were normalized to β-actin levels.

### Confocal microscopy

Rat and mice AM were seeded at 1×10^5^ and 1×10^4^ cells/well on 8-well Lab-Tek chamber slides and allowed to adhere for 90 min at 37°C in a 5% CO_2_ atmosphere. After treatment, the cells were fixed with ice-cold (−20°C) methanol for 2 min, washed three times with PBS, followed by permeabilization with 0.25% Triton X-100 for 7 min. Subsequently, cells were blocked with 10% BSA/PBS for 30 min and then incubated with anti-TLR4, anti-EEA1, anti-Vti1b, or control IgG at a 1∶60 dilution. Alexa Fluor 488 donkey anti-goat IgG, Alexa Fluor 633 goat anti-rabbit and donkey anti-mouse IgG served as secondary Abs at a 1∶500 dilution. Samples were analyzed using a Leica TCS SP confocal laser scanning microscope (Leica Microsystems, Bensheim, Germany) and images were assembled using Adobe Photoshop 10.0.

### Quantification of TLR4 expression and co-localization

All images used for quantification were scanned at the same pinhole, offset gain, and amplifier values below pixel saturation. Analysis was performed of at least two independent experiments with over 1×10^4^ to 1×10^5^ cells per condition, in which over 80% of the cells had similar staining patterns. TLR4 expression in IF pictures was determined by measuring the mean pixel intensity using ImageJ (NIH), and values are expressed as percentage change in mean pixel intensity = (treatment group intensity - control group intensity)/control group intensity ×100). Double-stained images used for co-localization analysis were obtained by sequential scanning for each channel to eliminate “cross-talk” of the channels and to ensure reliable quantification of co-localization. Quantitative analysis was performed using LAS-AF image analysis or co-localization plug in ImageJ. Threshold and background corrections were set based on Red-Green scatter gram. After setting background and threshold % co-localization was calculated.

### Measurement of TNF-α

TNF-α concentrations in BAL fluid and serum from *in vivo*-treated β-arrestin2^−/−^ mice and corresponding WT mice were determined by ELISA using a commercial kit specific for murine TNF-α (BD Biosciences), according to the manufacturer's protocol. Briefly, BAL samples were diluted 1∶2, 1∶4, and 1∶8 in the diluent provided with the assay. The range of detection was 31–2000 pg/ml. All plates were read on a microplate reader (Tecan, Crailsheim, Germany) and analyzed with the use of a computer-assisted analysis program (Magellan, Tecan).

### Statistical analysis

Data were statistically analyzed as indicated in the figure legends using GraphPad Prism (version 5.0; GraphPad). Values were considered significant when *p<*0.05. Data are presented as mean ± SEM.

## Results

### SP-A inhibits LPS-enhanced TLR4 expression in primary AM

**Figure 1:**
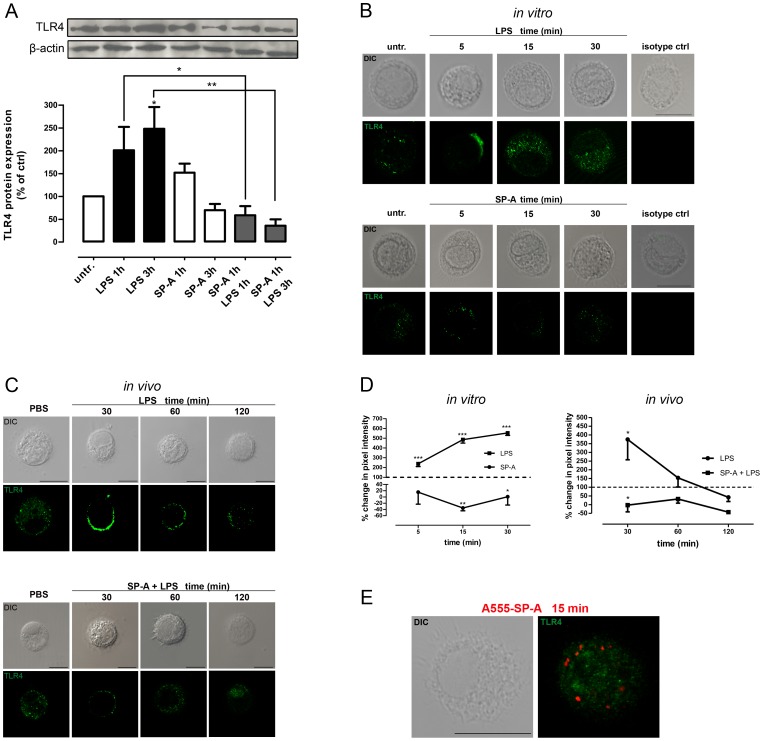
SP-A inhibits LPS-enhanced TLR4 expression in AM. A, Western blot of total TLR4 protein expression in primary rat AM treated with LPS (100 ng/ml, 1 h and 3 h), SP-A (40 µg/ml, 1 h and 3 h), or both (SP-A 1 h plus LPS 1 h or 3 h) as indicated. Equal amounts of whole cell lysates were subjected to SDS-PAGE and immunoblotted for TLR4 and β-actin. Data of at least five independent experiments were normalized to β-actin, basal TLR4 expression in untreated cells was set 100%, and calculated data were statistically analyzed by two-way ANOVA with Bonferroni's post test (mean ± SEM). *p<0.05; **p<0.01. B, Representative IF for TLR4 in LPS- and SP-A-treated primary rat AM. TLR4 distribution was monitored in cells treated with LPS (100 ng/ml) or SP-A (40 µg/ml) for the times indicated. Images are representative of at least two independent experiments in which over 80% of the cells had similar staining patterns. Scale bars, 10 µm. Isotype controls are also shown. Upper panel, LPS-treated cells. Lower panel, SP-A-treated cells. C, Representative IF for TLR4 in BAL cells from WT mice after intratracheal administration of 2.5 µg/kg BW of LPS or 5 mg/kg of SP-A plus 2.5 µg/kg of LPS. Images are representative of at least two independent experiments in which over 80% of the cells had similar staining patterns. Scale bars, 10 µm. D, Quantification of pixel intensity from confocal microscopy images stained for TLR4. Values are expressed as percentage change in mean pixel intensity ± SEM of one experiment with at least 20 cells per condition. Untreated control was set to 100% (dotted line) and data were statistically analyzed by one-way ANOVA with Bonferroni's posttest. *p<0.05; **p<0.01, ***p<0.001. E, Representative IF for TLR4 in primary rat AM treated A555-labeled SP-A for 15 min. Images are representative of two independent experiments in which over 80% of the cells had similar staining patterns. Scale bars, 10 µm.

LPS-induced TLR4 activation and signalling is regulated *via* distinct mechanisms including the cell surface expression of TLR4 which is in turn determined by the amount of TLR4 trafficking from the Golgi to the plasma membrane and the amount of TLR4 internalized into endosomes [5]. Therefore, we initially examined total TLR4 protein expression by Western analysis and the cellular positioning of TLR4 by confocal microscopy in SP-A- and LPS-treated primary rat and mouse AM *in vitro* and *in vivo*. Western kinetics revealed that incubation of rat AM with LPS (100 ng/ml) resulted in a 201±51% and 248±48% (p<0.05) enhancement of TLR4 protein expression after 1 h and 3 h, respectively. Pretreatment of AM with SP-A (40 µg/ml, 1 h) prior to LPS abrogated the LPS-enhanced TLR4 protein expression in these cells (Fig. 1A).

Subsequently performed confocal microscopy confirmed the data from Western analysis showing a significant increase in TLR4 staining over time for up to 30 min of LPS treatment (p<0.001) (Fig. 1B, upper panel and 1D, left panel). Furthermore, the confocal images indicate that LPS induced the recruitment of TLR4 to the cell periphery at very early time points (Fig. 1B, upper panel). In contrast, SP-A transiently decreased the TLR4 staining after 15 min (p<0.01) and 30 min (p<0.05) (Fig. 1B, lower panel and 1D, left panel) but had no effect on TLR4 localization compared to untreated cells (Fig. 1B, lower panel).

*In vivo* studies performed on BAL cells from WT mice treated with LPS (2.5 µg/kg BW) or SP-A (5 mg/kg BW) plus LPS (2.5 µg/kg BW) revealed that TLR4 staining was significantly enhanced after 30 min of LPS challenge (p<0.05) and declined after 60 and 120 min (Fig. 1C, upper panel and Fig. 1D, right panel). Treatment of the mice with SP-A plus LPS inhibited TLR4 staining after 30 min (p<0.05).

To determine whether SP-A directly interacts with TLR4, additional confocal microscopy was performed using Alexa Fluor 555-labeled SP-A. In primary rat AM, SP-A did not co-localize with TLR4 (Fig. 1E) within 15 min of incubation.

### LPS-induced co-localization of TLR4 with EEA 1 in primary AM is reduced by SP-A

**Figure 2:**
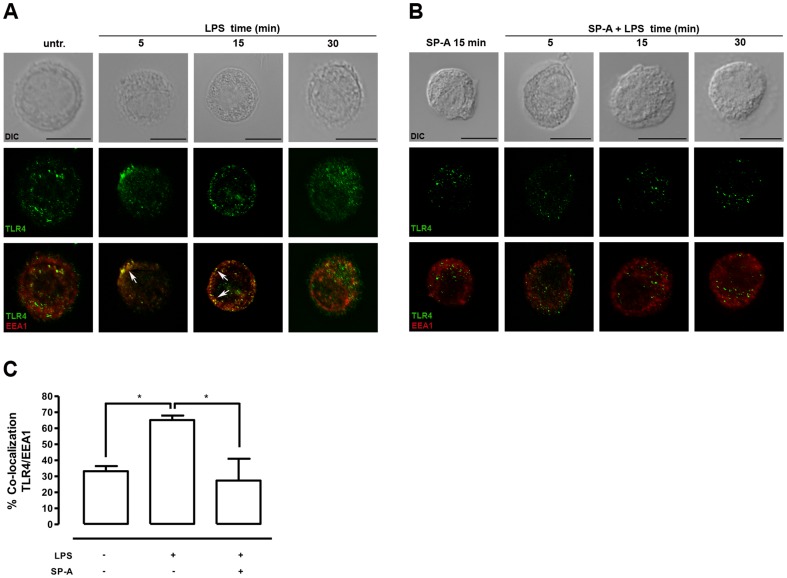
SP-A inhibits the LPS-induced co-localization of TLR4/EEA 1 in rat AM. A and B, Representative IF for TLR4 localization in primary rat AM treated with LPS (100 ng/ml), SP-A (40 µg/ml), or both as indicated. Images are representative of at least three independent experiments in which over 80% of the cells had similar staining patterns. Early endosomes were stained with EEA1. Arrows indicate the area of co-localization. Upper panels, DIC image. Middle panels, TLR4 staining. Lower panels, overlay of single stainings. Scale bars, 10 µm. C, Analysis of TLR4 and EEA1 co-localization. Values are expressed as percentage ± SEM of at least two independent experiments with at least 20 cells per condition. Data were statistically analyzed by one-way ANOVA with Bonferroni's posttest. *p<0.05.

The subcellular localization of TLR4 in primary rat AM was assessed by kinetic confocal microscopy studies using markers for different intracellular compartments. Treatment of the cells with isolated LPS (100 ng/ml) induced a rapid and significant (p<0.05) co-localization of TLR4 with the specific early endosome marker EEA1 after 5 min of incubation (Fig. 2A, 2C). The LPS-induced co-localization of TLR4/EEA1 was most evident for the first 15 min of incubation and decreased after 30 min. These data confirm and extend prior evidence for LPS-induced TLR4 translocation in HEK293 cells and human monocytes to primary AM [7], [9]. The role of SP-A in LPS-induced subcellular positioning of TLR4 was subsequently analyzed after treatment of the cells with SP-A, or SP-A plus LPS (Fig. 2B). Pretreatment of AM with SP-A (40 µg/ml, 15 min) prior to LPS (100 ng/ml, 5 min) abolished (p<0.05) the LPS-induced co-localization of TLR4 with EEA1 (Fig. 2B and 2C). The combined data indicate that SP-A modulates the subcellular positioning of TLR4 in LPS-stimulated primary rat AM.

### In β-arrestin 2^−/−^ AM SP-A fails to inhibit the LPS-induced co-localization of TLR4 with EEA1

**Figure 3:**
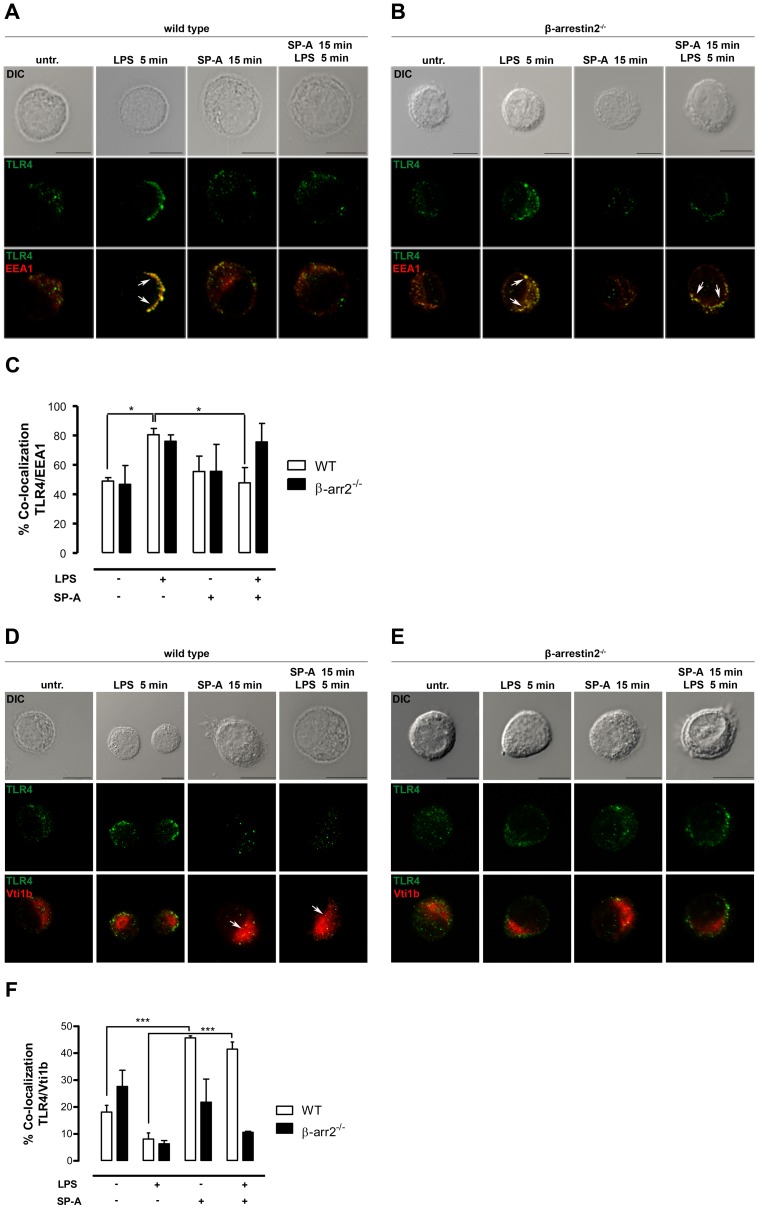
SP-A fails to inhibit the LPS-induced co-loclization of TLR4/EEA1 in β-arrestin 2^−/−^ AM. A + B and D + E, Representative IF for TLR4 localization in primary AM from WT (A and D) and β-arrestin 2^−/−^ mice (B and E) treated with LPS (100 ng/ml), SP-A (40 µg/ml), or both as indicated. Images are representative of at least three independent experiments in which over 80% of the cells had similar staining patterns. Early endosomes were stained with EEA1 and the post-Golgi compartment was stained with Vti1b. Arrows indicate the area of co-localization. Scale bars, 10 µm. C and F, Analysis of TLR4 and EEA (C) or TLR4 and Vti1b (F) co-localization. Values are expressed in percentage ± SEM of at least three independent experiments with at least 50 cells per condition. Data were statistically analyzed by one-way ANOVA with Bonferroni's posttest. *p<0.05; ***p<0.001.

The pleiotropic scaffold protein β-arrestin 2 has been described to play a critical role as negative regulator of LPS-induced TLR4 signaling [24]. Therefore, we next addressed the role of β-arrestin 2 in SP-A-mediated effects on LPS-induced TLR4 trafficking in primary AM from WT and β-arrestin 2^−/−^ mice. Data obtained from confocal analyses and subsequently performed quantification of co-localization revealed that the LPS-enhanced co-localization of TLR4/EEA1 in AM from WT mice was significantly reduced after pretreatment of the cells with SP-A (p<0.05) (Fig. 3A and 3C), confirming the data obtained from rat AM. In contrast, SP-A failed to inhibit the LPS-induced co-localization of TLR4 with EEA1 in β-arrestin 2^−/−^ AM (Fig. 3B and 3C). Additionally performed confocal analyses using the post-Golgi compartment marker Vti1b and subsequent quantitative co-localization analyses showed that SP-A enhanced the co-localization of TLR4 with the post Golgi compartment in WT AM (p<0.001) but failed to promote the co-localization of TLR4 and Vti1b in β-arrestin 2^−/−^ AM (Fig. 3D–F). The combined data suggest that the SP-A-modulated subcellular distribution of TLR4 in primary AM involves β-arrestin 2.

### SP-A enhances β-arrestin 2 protein expression in primary rat AM

**Figure 4:**
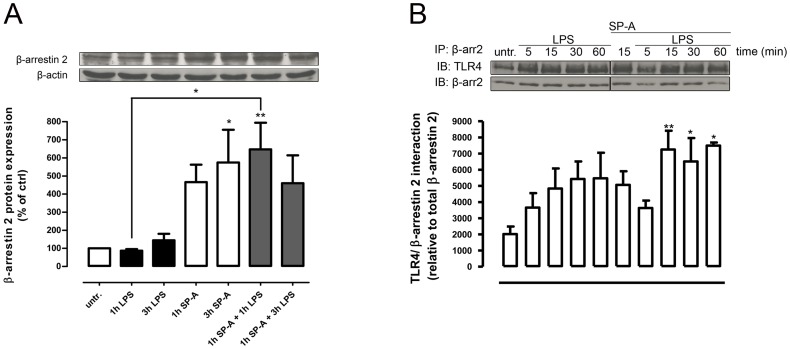
SP-A enhances β-arrestin 2 protein expression and LPS-induced β-arrestin 2/TLR4 interaction in primary AM. A, Western blot of total β-arrestin 2 protein expression in primary rat AM treated with LPS (100 ng/ml, 1 h and 3 h), SP-A (40 µg/ml, 1 h and 3 h) or both (SP-A 1 h plus LPS 1 h or 3 h) as indicated. Equal amounts of whole cell lysates were subjected to SDS-PAGE and immunoblotted for β-arrestin 2 and β-actin. Data of at least four independent experiments were normalized to β-actin, basal β-arrestin 2 expression in untreated cells was set 100%, and calculated data were statistically analyzed by two-way ANOVA with Bonferroni's post test (mean ± SEM). *p<0.05; **p<0.01. B, Immunoprecipitation (IP) of β-arrestin 2 and TLR4 immunoblot (IB) from rat AM lysates treated with LPS (100 ng/ml), SP-A (40 µg/ml), or both as indicated. Data of seven independent experiments were analyzed by one-way Anova with Dunett's posttest (mean ± SEM). *p<0.05; **p<0.01 (versus untreated control).

To determine whether SP-A directly affects β-arrestin 2 protein expression, we performed Western kinetics on primary rat AM. Exposure to SP-A, significantly enhanced β-arrestin 2 protein expression in a time-dependent manner (Fig. 4A). At 3 h of SP-A β-arrestin 2 expression had increased 6-fold (p<0.05) compared to untreated controls. In contrast, LPS stimulation of AM did not affect the expression of β-arrestin 2. Pretreatment of the cells with SP-A for 1 hr prior to LPS significantly increased β-arrestin 2 protein expression compared to LPS alone (p<0.05) (Fig. 4A). These results support a role for SP-A in the up-regulation of β-arrestin 2 expression in primary AM both under basal conditions and in the presence of LPS.

### SP-A enhances both basal and LPS-induced β-arrestin 2/TLR4 interaction in primary AM

We next asked whether SP-A can modify TLR4 positioning by integrating β-arrestin 2 scaffolding or signaling interactions. Co-immunoprecipitation experiments on primary rat AM showed that LPS increases β-arrestin 2/TLR4 association in a time-dependent manner within 60 min (Fig. 4B). Interestingly, treatment of the cells with SP-A alone also enhanced β-arrestin 2/TLR4 interaction, and further increased β-arrestin 2/TLR4 association when added to the cells 15 min prior to LPS (p<0.05 and p<0.001) (Fig. 4B). The data suggest that β-arrestin 2/TLR4 interaction in primary AM is enhanced by SP-A both constitutively and in the presence of LPS.

### The basal β-arrestin 2 protein expression in primary AM from SP-A−/− mice is significantly reduced and is rescued by SP-A

**Figure 5:**
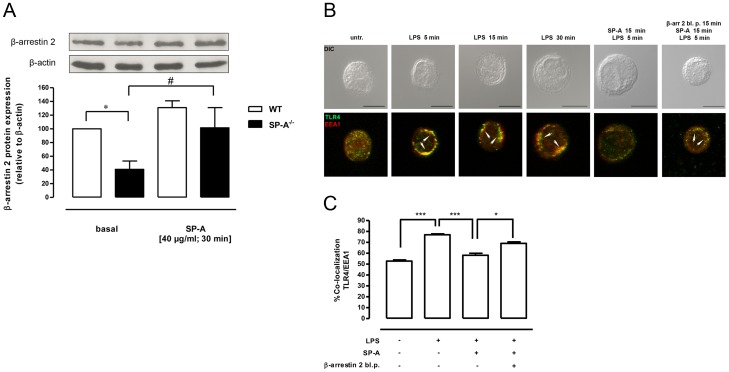
Reduced β-arrestin 2 expression and prolonged TLR4/EEA1 co-localization in SP-A^−/−^ AM are rescued by SP-A. A, Western blot of total β-arrestin 2 protein expression in primary AM from WT and SP-A^−/−^ mice treated with SP-A (40 µg/ml; 30 min). Equal amounts of whole cell lysates were subjected to SDS-PAGE and immunoblotted for β-arrestin 2 and β-actin. Data of four independent experiments were normalized to β-actin, basal β-arrestin 2 expression in WT mice was set 100%, and calculated data were statistically analyzed by one-way ANOVA with Bonferroni's posttest (mean ± SEM). *p<0.05 (WT versus SP-A^−/−^); ^#^p<0.05 (SP-A-treated versus basal). B, Representative IF for TLR4 localization in primary AM from SP-A^−/−^ mice treated with LPS (100 ng/ml), SP-A (40 µg/ml), LPS plus SP-A, or pre-treated with cell permeable β-arrestin 2 blocking peptides (15 min, 20 µg/ml) prior to LPS plus SP-A. Images are representative of two independent experiments in which over 80% of the cells had similar staining patterns. Early endosomes were stained with EEA1. Arrows indicate the area of co-localization. Upper panels, DIC image. Lower panels, overlay of single stainings. Scale bars, 10 µm. C, Analysis of TLR4 and EEA1 co-localization after treatment with LPS, SP-A plus LPS, or pretreatment with cell permeable β-arrestin 2 blocking peptides prior to LPS plus SP-A. Values are expressed as percentage ± SEM of two independent experiments with 20 cells per condition. Data were statistically analyzed by one-way ANOVA with Bonferroni's posttest. *p<0.05; ***p<0.001.

Mice that lack SP-A are more susceptible to LPS-induced inflammatory responses than WT mice [34], [35]. The LPS-induced increase in TNF-α, macrophage inflammatory protein-2, and nitric oxide in the BAL of SP-A^−/−^ mice can be restored by intratracheal administration of SP-A [34]. Furthermore SP-A^−/−^ pups show increased mortality after LPS inhalation and the survival of these mice can be improved through SP-A delivery by mouth [35]. In the present study we found that the constitutive β-arrestin 2 protein expression in primary AM from SP-A^−/−^ mice is significantly reduced compared to strain-matched WT mice (p<0.05) (Fig. 5A). The addition of exogenous SP-A rescued the basal low β-arrestin 2 protein expression in AM from SP-A^−/−^ mice (p<0.05) (Fig. 5A) suggesting that β-arrestin 2 is upregulated by SP-A in these cells. To determine whether the reduced β-arrestin 2 protein expression in AM from SP-A^−/−^ mice is associated with abnormal intracellular TLR4 trafficking, kinetic confocal microscopy studies after LPS treatment were performed. LPS significantly enhanced TLR4/EEA1 co-localization for up to 30 min (p<0.001) (Fig. 5B and C), indicating a prolonged TLR4 positioning at early endosomes in AM from SP-A^−/−^ mice. Treatment of the cells with both SP-A and LPS significantly inhibited the LPS-induced TLR4/EEA1 co-localization (p<0.001) (Fig. 5B and C). To further clarify the role of β-arrestin 2 in SP-A-mediated inhibition of LPS-induced TLR4/EEA1 co-localization, AM from SP-A^−/−^ mice were treated with cell permeable β-arrestin 2 blocking peptides prior to the addition of SP-A and LPS. Pretreatment of the cells with arrestin 2 blocking peptides antagonized the inhibitory effect of SP-A on LPS-induced TLR4/EEA1 co-localization (p<0.05) (Fig. 5B and C) suggesting that β-arrestin 2 is critically involved in this process.

### The lack of β-arrestin 2 enhances pulmonary LPS responsiveness *in vivo*

**Figure 6:**
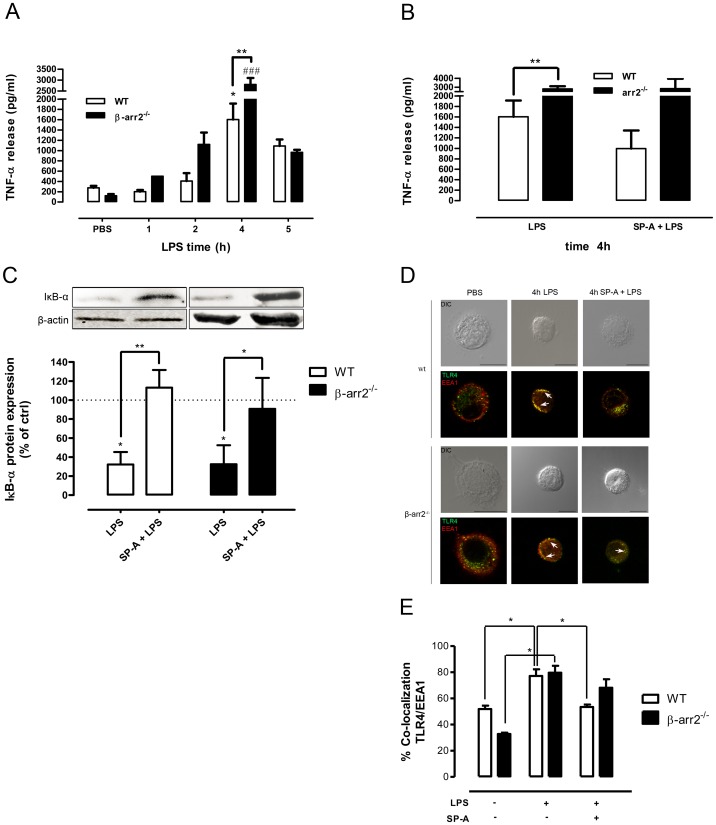
SP-A fails to inhibit enhanced LPS responsiveness in β-arrestin 2^−/−^ mice. A and B, TNF-α release in BAL fluid obtained from WT (n = 4–5) and β-arrestin 2^−/−^ mice (n = 3–5) at different times after intratracheal administration of 2.5 µg/kg BW of LPS or 5 mg/kg of SP-A plus 2.5 µg/kg of LPS. The control group received LPS-free PBS. *p<0.05 (LPS- versus PBS-treated WT mice); ^###^ p<0.001 (LPS-versus PBS-treated β-arrestin 2^−/−^ mice); **p<0.01 (LPS-treated WT mice versus LPS-treated β-arrestin 2^−/−^ mice). C, Western blot of cytosolic IκB-α protein expression in BAL cells from WT and β-arrestin 2^−/−^ mice 4 h after intratracheal administration of LPS or SP-A plus 2.5 µg/kg of LPS. Equal amounts of cytosolic fractions were subjected to SDS-PAGE and immunoblotted for IκB-α and β-actin. Data of three independent experiments were normalized to β-actin, basal IκB-α expression was set 100%, and calculated data were statistically analyzed by one-way ANOVA with Bonferroni's posttest. *p<0.05; **p<0.01. D, Representative IF for TLR4 localization in BAL cells from WT and β-arrestin 2^−/−^ mice after intratracheal administration of LPS or SP-A plus LPS. Images are representative of at least two independent experiments in which over 80% of the cells had similar staining patterns. Early endosomes were stained with EEA1. Arrows indicate the area of co-localization. Scale bars, 10 µm. E, Analysis of TLR4 and EEA1 co-localization. Values are expressed as percentage ± SEM of two independent experiments with at least 20 cells per condition. Data were statistically analyzed by one-way ANOVA with Bonferroni's posttest. *p<0.05.

Although there is strong evidence for cell and environment specificity of β-arrestin mode of action [19], a pulmonary LPS model in selectively β-arrestin 2^−/−^ mice has not been investigated so far. To determine the *in vivo* pulmonary LPS responsiveness, β-arrestin 2^−/−^ and WT mice were intratracheally challenged with LPS (2.5 µg/kg BW) or PBS and sacrified after 1, 2, 4, and 5 h. Within this time frame, no significant differences in lung TNF-α levels were seen between genotypes given PBS. LPS treatment enhanced TNF-α release in BAL fluid from both WT and β-arrestin 2^−/−^ mice in a time-dependent manner with a peak 4 h after challenge and declined after 5 h (Fig. 6A). LPS-induced TNF-α release in BAL fluid from β-arrestin 2^−/−^ mice was accelerated and significantly more pronounced than in WT mice (p<0.01) suggesting that the lack of β-arrestin 2 enhances lung susceptibility to LPS. A systemic TNF-α response was neither detected in β-arrestin 2^−/−^ mice nor in WT mice. Similarities in the phenotype of pro-inflammatory pulmonary responses between SP-A^−/−^ mice and β-arrestin 2^−/−^ mice support the hypothesis that the lack of SP-A influences lung inflammation partly mediated by inhibition of β-arrestin 2 expression.

### In β-arrestin 2-deficient mice, exogenous SP-A fails to inhibit LPS-induced TNF-α release and TLR4/EEA1 co-localization

To determine whether SP-A-mediated inhibition of LPS-induced TNF-α release engages on β-arrestin 2 *in vivo*, BAL fluid from β-arrestin 2^−/−^ mice and WT mice was analyzed for TNF-α concentrations 4 and 5 h after intratracheal application of SP-A (5 mg/kg) plus LPS (2.5 µg/kg). Whereas SP-A inhibited the LPS-induced TNF-α release in BAL fluid from WT mice by approximately 40%, SP-A failed to inhibit LPS-induced TNF-α release in BAL fluid from β-arrestin 2^−/−^ mice (Fig. 6B). LPS-induced TNF-α release depends on NF-κB activation. Since we [29], [36] and others [37] have shown that SP-A inhibits LPS-induced NF-κB activation *via* a promoted stabilization of its pivotal inhibitor IκB-α, we subsequently analyzed cytosolic IκB-α protein expression in BAL cells from WT and β-arrestin 2^−/−^ mice treated with PBS, LPS, or LPS plus SP-A for 4 h. SP-A antagonized LPS-induced degradation of IκB-α in both WT mice (p<0.01) and β-arrestin 2^−/−^ mice (p<0.05) (Fig. 6C), indicating that β-arrestin 2 is not involved in SP-A-mediated stabilization of IκB-α.

We next assessed the *in vivo* role of β-arrestin 2 in SP-A-mediated modulation of LPS-induced TLR4 trafficking. Confocal analysis of AM from *in vivo* LPS challenged β-arrestin2^−/−^ mice showed that LPS significantly enhanced the co-localization of TLR4 with EEA1 at 4 h (p<0.05) (Fig. 6E). However, compared to AM from WT mice, intratracheal co-administration of SP-A and LPS failed to inhibit TLR4 endosome positioning in AM from β-arrestin 2^−/−^ mice (Fig. 6D and 6E). The combined data indicate that SP-A-mediated inhibition of LPS-induced TLR4 signaling at both the level of TLR4 trafficking and TNF-α release *in vivo* critically involves β-arrestin 2.

## Discussion

The soluble C-type lectin SP-A is the most abundant surfactant-associated protein in the lung with important functions in pulmonary homeostasis *in vivo* [1]–[3]. SP-A^−/−^ mice exhibit increased susceptibility to infection, reduced survival, reduced macrophage activation, and inability to combat infection [38] after pulmonary challenge with a variety of clinically relevant pathogens, as well as to isolated LPS [34], [35]. Human lung diseases caused by Gram-negative bacteria and their invariant virulence factor LPS are a leading cause of mortality from infection [39]. Cellular responses to LPS are partly regulated through subcellular localization of TLR4 [5]. The pleiotropic scaffold protein β-arrestin 2 downregulates LPS-induced TLR4 signaling *in vivo* and *in vitro* [24]–[26], [40]. In this study, we demonstrate that SP-A inhibits LPS-induced TLR4 signaling by modulating TLR4 trafficking in primary AM. *In vivo* studies reveal that β-arrestin 2 serves as a mediator of SP-A-prevailed TLR4 positioning and attenuation of TLR4 signaling.

Confirming and extending recent data [41], [42], we found an up-regulation of TLR4 protein levels in primary rat AM within three hours of exposure to LPS. Pretreatment of the cells with SP-A, however, antagonized LPS-enhanced TLR4 expression in primary AM. Analysis of pixel intensity reveal that SP-A alone transiently decreased TLR4 staining at early time points (15 min and 30 min) of exposure. Recent data demonstrated that SP-A does not affect constitutive TLR4 surface expression on five-day old human monocyte-derived macrophages as determined by flow cytometry. Likewise under basal conditions, SP-A had very little effect on TLR4 mRNA expression during monocyte differentiation into macrophages [43]. The combined data would suggest that SP-A, under resting conditions, only transiently affects TLR4 abundancy, but can persistently decrease TLR4 expression levels in the presence of LPS. However, the mechanisms involved are unknown.

Confocal microscopy data presented here provide evidence that SP-A does not co-localize with TLR4 in primary AM. These data differ from a previous study demonstrating binding of SP-A to the recombinant extracellular domain of TLR4 employing microtiter binding assays and surface plasmon resonance analysis [15]. The divergent results could be due to the time points investigated (i.e., hours versus minutes) as well as the inherent limitations of both microtiter plate-based and cell-based *in vitro* binding assays.

In this study, LPS induced a rapid co-localization of TLR4 with EEA1-positive endosomes at very early time points in primary AM from rats and WT mice. In resting human monocytes and HEK cells, TLR4 cycles between the Golgi and at the plasma membrane and translocates to the cell surface upon LPS exposure [7], [9] in a MD2-dependent manner [44]. Upon LPS ligation, the LPS/TLR4/MD2 complex is internalized by endocytosis in early endosomes and is subsequently delivered to either recycling endosomes, TGN and Golgi for recycling, or lysosomes for degradation [6]–[9]. In the present study, confocal analysis show that pretreatment of primary AM with SP-A prior to LPS almost completely abolishes the LPS-induced TLR4/EEA1 positioning and promotes the co-localization of TLR4 with the post-Golgi compartment. These data suggest that SP-A may either decelerate the LPS-induced transport of TLR4 from the Golgi to the plasma membrane or accelerate the retrograde transport of TLR4 from endosomes to the Golgi. Since TLR4 is unable to transduce signaling from the Golgi compartment, the data strongly suggest that SP-A modifies TLR4 signaling *via* modulating TLR4 localization. In β-arrestin 2^−/−^ AM, however, SP-A fails to promote trans Golgi localization of TLR4 upon LPS stimulation *in vitro*, indicating that SP-A-modulated TLR4 positioning critically involves the presence of β-arrestin 2 and that β-arrestin 1 cannot functionally compensate for the lack of β-arrestin 2 in primary AM.

We found that LPS induced β-arrestin 2/TLR4 association in primary AM in a time-dependent manner, an effect that was further enhanced by pretreatment of the cells with SP-A. The lack of direct SP-A/TLR4 co-localization, the SP-A-mediated up-regulation of β-arrestin 2 protein expression and the SP-A-enhanced β-arrestin2/TLR4 interaction suggest that SP-A-modulated cellular distribution of TLR4 in primary AM is mediated indirectly by integrating β-arrestin 2 scaffolding interactions. The endocytic function of β-arrestin 2 is evident from its ability to bind to distinct receptors, AP-2 and clathrin [45]. In addition, β-arrestin 2 can serve as a sorting adaptor protein to mediate receptor entry into a degradative pathway [46]. The fact that both LPS [9] and SP-A [32], [47] are internalized by clathrin-mediated endocytosis raises the possibility that the constitutive internalization of endogenous SP-A by AM may be decisive for modulating LPS-induced TLR4 uptake, intracellular distribution, and signaling.

We found a significantly decreased protein expression of β-arrestin 2 in AM from SP-A^−/−^ mice that is restored to SP-A^+/+^ levels by the addition of SP-A suggesting that SP-A positively regulates β-arrestin 2. In addition, in AM from SP-A^−/−^ mice the localization of TLR4 in the endosome is markedly prolonged compared to its distribution in WT AM. Because AM are key to an appropriate innate immune response of the lung, abnormal TLR4 distribution in these cells may be one of the causes of the pro-inflammmatory phenotype of SP-A^−/−^ mice after pulmonary LPS challenge. The altered distribution of TLR4 in AM from SP-A^−/−^ mice is restored by the addition of SP-A and this effect is largely abolished by pretreatment of the cells with β-arrestin 2 blocking peptides. Together the findings suggest that SP-A participates in TLR4 signaling pathways by regulating TLR4 distribution *via* β-arrestin 2. However, it remains to be identified how β-arrestin 2-mediated distribution of TLR4 after SP-A stimulation is regulated.

We have previously shown that SP-A transiently enhances the expression of functionally active small GTPases Rab7 and Rab7b in primary AM [18]. The late endosome-associated Rab7 is a key regulator in lysosome-directed transport [48]. Rab7b is involved in downmodulation of TLR4 signaling [49] and is essential for the retrograde transport from endosomes to the trans-Golgi network [50]. Therefore it is possible that SP-A modifies TLR4 distribution *via* Rab7b and/or Rab7 and that redundancies in this regulation exist. Whether RabGTPases and β-arrestin 2 functionally affect each other is unknown, though proteomic analyses of proteins that interact with β-arrestins demonstrate a direct interaction of small GTPases-related proteins with β-arrestins [51].

A pulmonary LPS model in selectively β-arrestin 2^−/−^ mice has not been addressed so far. Our data show, that LPS-induced TNF-α release in BAL fluid from β-arrestin 2^−/−^ mice was significantly accelerated and more pronounced than in WT mice indicating that the lack of β-arrestin 2 enhances lung susceptibility to LPS in a time dependent manner. These data support previous *in vivo* studies demonstrating that intraperitoneally LPS-treated β-arrestin 2^−/−^ mice had higher levels of pro-inflammatory cytokines and were more susceptible to endotoxic shock [24]. In polymicrobial sepsis, the survival rate of β-arrestin 2^−/−^ mice is significantly decreased compared to WT mice and β-arrestin 2^−/−^ mice exhibit a more severe lung damage and higher bacterial loads than WT mice [21]. In addition, *in vitro* loss and gain of function studies in HEK/TLR4 cells demonstrate that β-arrestin 2 dampens LPS-induced NF-κB activation [40]. In contrast, another *in vivo* study showed that β-arrestin 2^−/−^ mice intraperitoneally challenged with LPS were protected from TLR4-mediated endotoxic shock and lethality in a gender-dependent manner *via* mechanisms entailing chromatin modifications [52]. However, using sex-mixed arrestin 2^−/−^ mice in our study, we could not observe gender-dependent protective effects on LPS susceptibility of the lung.

As we have shown here, SP-A failed to inhibit LPS-induced TNF-α release and TLR4 endosome positioning in β-arrestin 2^−/−^ mice indicating that β-arrestin 2 is engaged in the anti-inflammatory effects of SP-A *in vivo*. Confirming and extending previous data [29], [36], we could show that SP-A promoted the stabilization of the predominant NF-κB inhibitor IκB-α in both WT and β-arrestin 2^−/−^ mice after LPS challenge. Therefore, the failure of SP-A to inhibit NF-κB-dependent TNF-α release in β-arrestin 2^−/−^ mice is either independent of a mechanism involving the IκB-α negative feedback circuit or that this pathway is not operative in the absence of β-arrestin 2.

In conclusion, this study demonstrates that SP-A modulates the spatiotemporal compartmentalization and signalling of LPS-induced TLR4 *in vitro* and *in vivo* engaging β-arrestin 2. Further investigations on cell-type-specific TLR4 responses and the impact of tissue-specific factors on TLR4 positioning and thus ligand sensing will help to potentially improve the qualitative and quantitative outcome of innate immune responses of the lung to Gram-negative bacteria.

## References

[1] LeVine AM, Whitsett JA (2001) Pulmonary collectins and innate host defense of the lung. Microbes Infect 3: 161–166. 11251302 10.1016/s1286-4579(00)01363-0

[2] Wright JR (2005) Immunoregulatory functions of surfactant proteins. Nat Rev Immunol 5: 58–68. 15630429 10.1038/nri1528

[3] Chroneos ZC, Sever-Chroneos Z, Shepherd VL (2010) Pulmonary surfactant: an immunological perspective. Cell Physiol Biochem 25: 13–26. 20054141 10.1159/000272047 PMC3025886

[4] Kawai T, Akira S (2010) The role of pattern-recognition receptors in innate immunity: update on Toll-like receptors. Nat Immunol 11: 373–384. 20404851 10.1038/ni.1863

[5] McGettrick AF, O'Neill LA (2010) Localisation and trafficking of Toll-like receptors: an important mode of regulation. Curr Opin Immunol 22: 20–27. 20060278 10.1016/j.coi.2009.12.002

[6] Thieblemont N, Wright SD (1999) Transport of lipopolysaccharide to the golgi apparatus. J Exp Med 190: 523–534. 10449523 10.1084/jem.190.4.523 PMC2195609

[7] Latz E, Visintin A, Lien E, Fitzgerald KA, Monks BG, et al (2002) Lipopolysaccharide rapidly traffics to and from the Golgi apparatus with the toll-like receptor 4-MD-2-CD14 complex in a process that is distinct from the initiation of signal transduction. J Biol Chem 277: 47834–47843. 12324469 10.1074/jbc.M207873200

[8] Hornef MW, Frisan T, Vandewalle A, Normark S, Richter-Dahlfors A (2002) Toll-like receptor 4 resides in the Golgi apparatus and colocalizes with internalized lipopolysaccharide in intestinal epithelial cells. J Exp Med 195: 559–570. 11877479 10.1084/jem.20011788 PMC2193765

[9] Husebye H, Halaas O, Stenmark H, Tunheim G, Sandanger O, et al (2006) Endocytic pathways regulate Toll-like receptor 4 signaling and link innate and adaptive immunity. EMBO J 25: 683–692. 16467847 10.1038/sj.emboj.7600991 PMC1383569

[10] Barton GM, Kagan JC (2009) A cell biological view of Toll-like receptor function: regulation through compartmentalization. Nat Rev Immunol 9: 535–542. 19556980 10.1038/nri2587 PMC3934928

[11] Kagan JC (2010) Recycling endosomes and TLR signaling – the Rab11 GTPase leads the way. Immunity 33: 578–580. 21029967 10.1016/j.immuni.2010.10.003

[12] Stamme C, Müller M, Hamann L, Gutsmann T, Seydel U (2002) Surfactant protein A inhibits lipopolysaccharide (LPS)-induced immune cell activation by preventing the interaction of LPS with LPS-binding protein. Am J Respir Cell Mol Biol 27: 353–360. 12204898 10.1165/rcmb.4812

[13] Sano H, Sohman H, Muta T, Nomura S, Voelker DR, et al (1999) Pulmonary surfactant protein A modulates the cellular response to smooth and rough lipopolysaccharides by interation with CD14. J Immunol 63: 387–395. 10384140

[14] Sano H, Chiba H, Iwaki D, Sohma H, Voelker DR, et al (2000) Surfactant proteins A and D bind CD14 by different mechanisms. J Biol Chem 275: 22442–51. 10801802 10.1074/jbc.M001107200

[15] Yamada C, Sano H, Shimizu T, Mitsuzawa H, Nishitani C, et al (2006) Surfactant protein A directly interacts with TLR4 and MD-2, and regulates inflammatory cellular response: importance of supratrimeric oligomerzation. J Biol Chem 281: 21771–21780. 16754682 10.1074/jbc.M513041200

[16] Gardai SJ, Xiao YQ, Dickinson M, Nick JA, Voelker DR, et al (2003) By binding SIRPalpha or calreticulin/CD91, lung collectins act as dual function surveillance molecules to suppress or enhance inflammation. Cell 115: 13–23. 14531999 10.1016/s0092-8674(03)00758-x

[17] Alcorn JF, Wright JR (2004) Surfactant protein A inhibits alveolar macrophage cytokine production by CD14-independent pathway. Am J Physiol Lung Cell Mol Physiol 286: L129–L136. 12959932 10.1152/ajplung.00427.2002

[18] Sender V, Moulakakis C, Stamme C (2011) Pulmonary surfactant protein A enhances endolysosomal trafficking in alveolar macrophages through regulation of Rab7. J Immunol 186: 2397–2411. 21248257 10.4049/jimmunol.1002446

[19] Lattin JE, Greenwood KP, Daly NL, Kelly G, Zidar DA, et al (2009) Beta-arrestin 2 is required for complement C1q expression in macrophages and contrains factor-independet survival. Mol Immunol 47: 340–347. 19783052 10.1016/j.molimm.2009.09.012

[20] Zhang M, Liu X, Zhang Y, Zhao J (2010) Loss of beta-arrestin 1 and beta-arrestin 2 contributes to pulmonary hypoplasia and neonatal lethality in mice. Dev Biol 339: 407–417. 20060823 10.1016/j.ydbio.2009.12.042

[21] Fan H, Bitto A, Zingarelli B, Luttrell LM, Borg K, et al (2009) Beta-arrestin 2 negatively regulates sepsis-induced inflammation. Immunology 130: 344–351. 10.1111/j.1365-2567.2009.03185.x PMC2913214 20465566

[22] Walker JK, Fong AM, Lawson BL, Savov JD, Patel DD, et al (2003) β-arrestin-2 regulates the development of allergic asthma. J Clin Invest 112: 566–574. 12925697 10.1172/JCI17265 PMC171386

[23] Rajagopal S, Rajagopal K, Lefkowitz RJ (2010) Techaing old receptors new tricks: biasing seven-transmembrane receptors. Nat Rev Drug Discov 9: 373–386. 20431569 10.1038/nrd3024 PMC2902265

[24] Wang Y, Tang Y, Teng L, Wu Y, Zhao X, et al (2006) Association of beta-arrestin and TRAF6 negatively regulates Toll-like receptor-interleukin 1 receptor signalling. Nat Immunol 7: 139–147. 16378096 10.1038/ni1294

[25] Witherow DS, Garrison TR, Miller WE, Lefkowitz RJ (2004) beta-Arrestin inhibits NF-kappaB activity by means of its interaction with the NF-kappaB inhibitor IkappaBalpha. Proc Natl Acad Sci U S A 101: 8603–8607. 15173580 10.1073/pnas.0402851101 PMC423241

[26] Gao H, Sun Y, Wu Y, Luan B, Wang Y, et al (2004) Identification of beta-arrestin2 as a G protein-coupled receptor-stimulated regulator of NF-kappaB pathways. Mol Cell 14: 303–317. 15125834 10.1016/s1097-2765(04)00216-3

[27] Li G, Siddiqui J, Hendry M, Akiyama J, Edmondson J, et al (2002) Surfactant protein-A-deficient mice display an exaggerated early inflammatory response to a beta-resistant strain of influenza A virus. Am J Respir Cell Mol Biol 26: 277–282. 11867335 10.1165/ajrcmb.26.3.4584

[28] Bohn LM, Lefkowitz RJ, Gainetdinov RR, Peppel K, Caron MG, et al (1999) Enhanced morphine analgesia in mice lacking beta-arrestin 2. Science 286: 2495–2498. 10617462 10.1126/science.286.5449.2495

[29] Moulakakis C, Adam S, Seitzer U, Schromm AB, Leitges M, et al (2007) Surfactant protein A activation of atypical protein kinase C zeta in IkappaB-alpha-dependent anti-inflammatory immune regulation. J Immunol 179: 4480–4491. 17878344 10.4049/jimmunol.179.7.4480

[30] Galanos C, Lüderitz O (1975) Electrodialysis of lipopolysaccharides and their conversion to uniform salt forms. Eur J Biochem 54: 603–610. 1100380 10.1111/j.1432-1033.1975.tb04172.x

[31] Wright JR, Wager RE, Hawgood S, Dobbs L, Clements JA (1987) Surfactant apoprotein Mr = 26,000-36,000 enhances uptake of liposomes by type II cells. J Biol Chem 262: 2888–2895. 3818626

[32] Moulakakis C, Stamme C (2009) Role of clathrin.mediated endocytosis of surfactant protein A by alveolar macrophages in intracellular signalling. Am J Cell Mol Physiol 296: L430–L441. 10.1152/ajplung.90458.2008 19136579

[33] Stamme C, Walsh E, Wright JR (2000) Surfactant protein A differentially regulates IFN-γ and LPS-induced nitrite production by rat alveolar macrophages. Am J Cell Mol Biol 23: 772–779. 10.1165/ajrcmb.23.6.4083 11104730

[34] Borron P, McIntosh JC, Korfhagen JA, Taylor J, Wright JR (2000) Surfactant-associated protein A inhibits LPS-induced cytokine and nitric oxide production in vivo. Am J Physiol Lung Cell Mol Physiol 278: L840–847. 10749762 10.1152/ajplung.2000.278.4.L840

[35] George CL, Goss KL, Meyerholz DK, Lamb FS, Snyder JM (2008) Surfactant-associated protein A provides critical immunoprotection in neonatal mice. Infect Immun 76: 380–390. 17967856 10.1128/IAI.01043-07 PMC2223658

[36] Wu Y, Adam S, Hamann L, Heine H, Ulmer AJ, et al (2004) Accumulation of inhibitory kappaB-alpha as a mechanism contributing to the anti-inflammatory effects of surfactant protein-A. Am J Respir Cell Mol Biol 31: 587–594. 15308505 10.1165/rcmb.2004-0003OC

[37] Ramadas RA, Wu L, LeVine AM (2009) Surfactant protein A enhances production of secretory leukoprotease inhibitor and protects it from cleavage by matrix metalloproteinase. J Immunol 182: 1560–1567. 19155504 10.4049/jimmunol.182.3.1560

[38] LeVine AM, Whittsett JA, Gwozdz JA, Richardson TR, Fisher JH, et al (2000) Distinct effects of surfactant protein A or D deficiency during bacterial infection on the lung. J Immunol 165: 3934–3940. 11034401 10.4049/jimmunol.165.7.3934

[39] World Health Organization (2004) The global burden of disease: 2004 update [Accessed 2010] Geneva.

[40] Fan H, Luttrell LM, Tempel GE, Senn JJ, Halushka PV, et al (2007) Beta-arrestin 1 and 2 differentially regulate LPS-induced signaling and pro-inflammatory gene expression. Mol Immunol 44: 3092–3099. 17418896 10.1016/j.molimm.2007.02.009 PMC1945129

[41] Juarez E, Nuñez C, Sada E, Ellner JJ, Schwander SK, et al (2010) Differential expression of Toll-like receptors on human alveolar macrophages and autologous peripheral monocytes. Respir Res 11: 2. 20051129 10.1186/1465-9921-11-2 PMC2817655

[42] Thorley AJ, Grandolfo D, Lim E, Goldstraw P, Young A, et al (2011) Innate immune response to bacterial ligands in the peripheral human lung- role of alveolar epithelial TLR expression and signalling. PLoS One 6: e21827. 21789185 10.1371/journal.pone.0021827 PMC3137597

[43] Henning LN, Azad AK, Parsa KV, Crowther JE, Tridandapani S, et al (2008) Pulmonary surfactant protein A regulates TLR expression and activity in human macrophages. J Immunol 180: 7847–7858. 18523248 10.4049/jimmunol.180.12.7847 PMC2562757

[44] Nagai Y, Akashi S, Nagafuku M, Ogata M, Iwakura Y, et al (2002) Essential role of MD-2 in LPS responsiveness and TLR4 distribution. Nat Immunol 3: 667–672. 12055629 10.1038/ni809

[45] DeFea KA (2011) Beta-arrestins as regulators of signal termination and transduction: How do they determine what to scaffold ? Cellular Signal 23: 621–629. 10.1016/j.cellsig.2010.10.004 20946952

[46] Bhandari D, Trejo J, Benovic JL, Marchese A (2007) Arrestin-2 interacts with the ubiquitin-protein isopeptide ligase atrophin-interacting protein 4 and mediates endosomal sorting of the chemokine receptor CXCR4. J Biol Chem 282: 36971–36979. 17947233 10.1074/jbc.M705085200

[47] Crowther JE, Schlesinger LS (2006) Endocytic pathway for surfactant protein A in human macrophages: binding, clathrin-mediated uptake, and trafficking through the endolysosomal pathway. Am J Physiol Lung Cell Mol Physiol 290: L334–L342. 16169899 10.1152/ajplung.00267.2005

[48] Bucci C, Thomsen P, Nicoziani P, McCarthy J, van Deurs B (2000) Rab7: a key to lysosome biogenesis. Mol Biol Cell 11: 467–480. 10679007 10.1091/mbc.11.2.467 PMC14786

[49] Wang Y, Chen T, Han C, He D, Liu H, et al (2007) Lysosome-associated small Rab GTPase Rab7b negatively regulates TLR4 signaling in macrophages by promoting lysosomal degradation of TLR4. Blood 110: 952–971. 10.1182/blood-2007-01-066027 17395780

[50] Progida C, Cogli L, Piro F, De Luca A, Bakke O, et al (2010) Rab7b controls trafficking from endosomes to the TGN. J Cell Sci 123: 1480–1491. 20375062 10.1242/jcs.051474

[51] Xiao K, McClatchy DB, Shukla AK, Chen M, Shenoy SK, et al (2007) Functional specialization of β-arrestin interactions revealed by proteomic analysis. Proc Natl Acad Sci U S A 104: 12011–120. 17620599 10.1073/pnas.0704849104 PMC1913545

[52] Porter KJ, Gonipeta B, Parvataneni S, Appledorn DM, Patial S, et al (2010) Regulation of lipopolysaccharide-induced inflammatory response and endotoxemia by beta-arrestins. J Cell Physiol 225: 406–416. 20589830 10.1002/jcp.22289 PMC2930123

